# The Potential of Zebrafish as a Model Organism for Improving the Translation of Genetic Anticancer Nanomedicines

**DOI:** 10.3390/genes8120349

**Published:** 2017-11-28

**Authors:** Gutiérrez-Lovera C, Vázquez-Ríos AJ, Guerra-Varela J, Sánchez L, de la Fuente M

**Affiliations:** 1 Zoology, Genetics and Physical Anthropology Department Veterinary Faculty, Universidade de Santiago de Compostela, Lugo 27002, Spain; carlha.gutierrez@rai.usc.es (G.-L.C); jorge.guerra@usc.es (G.-V.J); lauraelena.sanchez@usc.es (S.L); 2 Nano-Oncology Unit, Translational Medical Oncology Group, Health Research Institute of Santiago de Compostela (IDIS), Clinical University Hospital of Santiago de Compostela (CHUS), CIBERONC, Santiago de Compostela 15706, Spain; abijudit.vazquez@rai.usc.es; 3 Geneaqua S.L., Lugo 27002, Spain

**Keywords:** nanomedicines, cancer, gene therapies, zebrafish, translation

## Abstract

In the last few decades, the field of nanomedicine applied to cancer has revolutionized cancer treatment: several nanoformulations have already reached the market and are routinely being used in the clinical practice. In the case of genetic nanomedicines, i.e., designed to deliver gene therapies to cancer cells for therapeutic purposes, advances have been less impressive. This is because of the many barriers that limit the access of the therapeutic nucleic acids to their target site, and the lack of models that would allow for an improvement in the understanding of how nanocarriers can be tailored to overcome them. Zebrafish has important advantages as a model species for the study of anticancer therapies, and have a lot to offer regarding the rational development of efficient delivery of genetic nanomedicines, and hence increasing the chances of their successful translation. This review aims to provide an overview of the recent advances in the development of genetic anticancer nanomedicines, and of the zebrafish models that stand as promising tools to shed light on their mechanisms of action and overall potential in oncology.

## 1. Nanotechnology Provides Innovative Approaches to Cancer Management

In recent decades, an increasing understanding of the molecular and biological basis of cancer and the discovery of novel technologies has led to improvements in cancer survival. The development of early detection tools and targeted treatments, as well as changes in patients’ lifestyle, have contributed to this higher rate of cancer survival. The development of new nanomedicines for cancer treatment is an interdisciplinary research field that includes biology, chemistry, engineering, and medicine, with a clear goal: advancing cancer detection, diagnosis, and treatment.

Different types of nanocarriers, including liposomes and other lipid-based nanosystems, polymer-based nanoparticles, micelles, polyplexes, dendrimers, polymersomes and drug/protein conjugates have been proposed during the last few decades in cancer research [1,2,3,4,5,6,7,8,9,10,11]. For cancer treatment, the goal is to enhance the efficacy and decrease the toxicity of the current therapeutics by altering their pharmacokinetic profile, increasing their solubility and stability in biological fluids, augmenting their accumulation in tumors, and reducing their toxicity. Biological drugs, such as gene therapies, peptides and proteins, can also benefit greatly from the application of nanotechnology that could protect them from premature degradation and facilitate their access to the intracellular compartment [12,13,14,15]. Liposomes are the most common type of nanostructure that have translated into marketed products [16,17,18,19,20,21]. Back in 1995, the US Food and Drug Administration (FDA) approved the first nanoparticle for cancer treatment, Doxil^©^, a liposomal nanoparticle loaded with the chemotherapeutic drug doxorubicin [22]. Since then, other nanotherapeutics based on liposomes have reached the market such as Pegylated liposomal doxorubicin (Doxil^©^/Caelyx^©^), liposomal cytarabine (DepoCyt^©^), Daunorubicin citrate Liposomes (DaunoXome^©^), liposomal doxorubicin (Myocet^©^), Vincristine Sulfate Liposomes (Marqibo^©^), liposomal irinotecan (Onivyde^©^). Paclitaxel polymeric nanoparticles (Opaxio^©^), pegylated L-asparaginase polymeric nanoparticles (Oncaspar^©^), leuprolide acetate polymeric micelles (Eligard^©^), oxaliplatin micelles (Eloxatin^©^), polymer–protein conjugate pegfilgrastim (Neulasta^©^), albumin-paclitaxel (Abraxane^©^), Denileukin diftitox (Ontak^©^), Brentuximab-Monomethyl auristatin E (MMAE) (Adcetris™), and Trastuzumab-Emtansine (Kadcyla^©^) are examples of different types of nanostructures that have led to products already in clinical use.

Apart from their use in the possible development of nanotherapeutics, nanoparticles are also useful tools in the diagnosis field, due, in the case of inorganic nanoparticles, to their intrinsic properties that allow a direct tracking, and, in the case of organic nanoparticles, to their ability to accommodate/encapsulate different molecules and contrast agents for imaging applications. Many contrast agents are currently being studied with this goal in mind, including super-paramagnetic iron oxide nanoparticles and ultra-small super-paramagnetic iron oxide nanoparticles, heavy metal (i.e., gold, lanthanide, and tantalum) nanoparticles, technetium-99m (^99m^TC) sulphur colloid nanoparticles, I-labeled cRGDY silica nanoparticles, surface-enhanced Raman scattering nanoparticles, and single-walled carbon nanotubes. Organic nanoparticles such as liposomes, micelles, and nanoemulsions can, for example, encapsulate super-paramagnetic iron oxide nanoparticles, or be radiolabeled with radioisotopes such as ^89^Zr, ^111^In, ^18^F, ^64^Cu or ^68^Ga for molecular imaging [23,24,25,26]. The imaging modalities currently available experimentally are: ultrasound, magnetic resonance imaging (MRI), optical imaging, molecular imaging, computed tomography (CT), positron emission tomography (PET), and single-photon emission computed tomography (SPECT). However, in clinics, the most used modalities for whole-body imaging are CT, MRI, PET and SPECT. For organ-specific examinations, ultrasounds are of preference since they are faster and less expensive, while, for superficial lesions, endoscopic, and intraoperative procedures, optical and photo-acoustic applications are more suitable [27,28].

Finally, nanoparticles also have a great potential as nanotheranostics, i.e., multifunctional nanoparticles that combine, into a single entity, elements for therapy and for diagnosis. Nanotheranostics have been explored for applications combining different imaging modalities and therapeutic applications, such as photodynamic therapy, photothermal, phototriggered chemotherapeutic release, ultrasound triggered, electro-thermal, magnetothermal, X-ray, and radiofrequency therapies [27,29]. Moreover, nanotheranostics are gathering great interest because they might provide a deeper understanding of key aspects that could make a nanoparticle formulation successful—such as drug release kinetics and penetration of nanocarriers within tumors—monitoring therapeutic responses, as well as allowing the implementation of novel strategies, such as imaging-guided local therapy [30,31]. To date, there is only one formulation undergoing clinical trials (Phase I) for the treatment of multiple brain metastases, AGuIX^®^ (Activation and Guidance of Irradiation by X-ray), a gadolinium-based nanoparticle of around 5 nm diameter, developed mainly for imaging applications due to its magnetic resonance contrast properties. However, when it is combined with X-ray radiation, it increases three-fold the radiotherapy effectiveness in mice, playing a double role, as radiosensitizer and as imaging agent (NCT02820454) [32,33]. We believe that nanotheranostics have a lot of potential in cancer management, and could definitively make an impact in the clinical practice by, concurrently, diagnosing the disease, helping patients stratification, guiding focal therapy, tracking drug release and penetration within tumors, monitoring response, and, if required, switching treatments.

## 2. Genetic Nanomedicines and the Main Challenges for Their Translation to the Clinic

Advances in genetics and molecular biology have led to the development of new therapies that can specifically modulate the expression of relevant genes in order to correct abnormalities and restore their original biological function. Some of the strategies of gene therapy include (i) silencing oncogene expression, (ii) promoting tumor-suppressor genes, (iii) correcting mutations, (iv) suicide gene therapy, (v) suppressing tumor angiogenesis, and (vi) activating an immune response against tumor cells. For these purposes, plasmid DNA (pDNA), minicircles (supercoiled circular DNA), oligonucleotides (ASOs, decoys, aptamers), RNA interference (short-hairpin (shRNA), small interfering RNA (siRNA) and microRNA (miRNA)) are being extensively explored [34]. However, because naked nucleic acids are vulnerable to enzymatic degradation, rapid clearance, and non-specific biodistribution, only low gene expression efficiencies can be achieved. Hence, the primary challenge of gene therapy is to develop effective carriers able to protect the nucleic acids and facilitate their internalization into the targeted cells at the targeted site [35].

Traditionally, vectors for gene therapy applications are divided into viral and non-viral carriers. Most gene vectors (~69%) currently undergoing clinical trials involve viruses (i.e., retroviruses, lentiviruses, adenoviruses, and adeno-associated viruses). In August 2017, the FDA approved the first gene therapy in the United States, Tisagenlecleucel (Kymriah^©^) from Novartis Pharma AG (Basel, Switzerland), for certain pediatric and young adult patients with a form of acute lymphoblastic leukemia whose first-line drugs have failed [36]. This pioneer gene therapy—based on a self-inactivating lentiviral vector that contains extensively modified sequences from HIV-1 so as to deliver chimeric antigen receptor (CAR)-encoding sequences into T cells to target and kill leukemia cells with specific antigen (CD19) on the surface—achieves an overall remission rate of 83% (52/63) in this patient population [37]. Despite these advances, many concerns still remain regarding the use of viral vectors, such as their potential immunogenicity, the possibility of reversion to the virulent form or the viruses, and also their high production costs [35]. Alternative synthetic vectors, made out of natural, semi-synthetic or synthetic materials, offer a safer alternative to introduce genetic materials into the targeted cells. Numerous non-viral gene delivery systems for different types of nucleic acids (mainly pDNA, siRNA and miRNA) have been described to date [34,38]. Different applications for the development of novel anticancer genetic nanomedicines have similarly being explored, including suicide gene therapies, anti-angiogenic gene therapies, immunotherapies, restoration of oncosuppressor RNAs, or gene silencing of oncogenes, or specific non-coding RNAs (antagomirs), or proteins involved in resistance to chemo- and radio-therapies, anti-apoptotic proteins, epigenetic regulation, etc., as recently reviewed by Bottai et al. [39]. The main preclinical studies of the different applications of nanoparticles for gene therapy reported successful in mice models are summarized in Table 1 (reporter genes and experiments referring to over expression/silencing of housekeeping genes are not included).

Recent advances in non-viral gene vectors regarding efficiency, specificity, safety and gene expression durability have led to an increase in the number of nanoparticle-based gene delivery vectors in clinical trials while the number of viral vectors have dropped significantly [53]. Some examples in cancer are related to liposomes for siRNA, microRNA or pDNA delivery (NCT01591356, NCT01829971, NCT01489371, NCT02340156); lipid nanoparticles (NCT02314052, NCT01437007) or polymeric nanoparticles (NCT02956317) [54]. Unfortunately, non-viral vectors have not reached the market yet.

The design of successful synthetic nanovectors poses a big challenge since they need to overcome important biological barriers. Nanovectors need (i) to be safe and adequate for parenteral administration, (ii) efficiently protect nucleic acids from degradation, and (iii) promote their access to the target intracellular compartment in the target cell (depending on the selected gene therapeutic system, i.e., plasmid DNA, RNAi, non-coding RNA (ncRNAs), oligonucleotides, etc.), in enough amounts to mediate a therapeutic effect (depending on the potency of the molecule, specificity, and stability) [34,55,56,57,58]. All these aspects should be taken into consideration from early development to increase the chances of translation into early-phase clinical trials [11,59,60,61]. The development of functional assays and the selection of adequate animal models for therapeutic evaluation are also key steps that critically affect the outcome of the preclinical evaluation.

Although a number of gene-delivery nanovectors have been claimed to be efficient, most of the studies have been done in vitro, on immortalized cancer cell lines, and only a few have actually addressed the therapeutic outcome in vivo. While in vitro experiments include evaluation of toxicity (e.g., MTT (3-(4,5-Dimethylthiazol-2-yl)-2,5-Diphenyltetrazolium Bromide), MTS (3-(4,5-dimethylthiazol-2-yl)-5-(3-carboxymethoxyphenyl)-2-(4-sulfophenyl)-2H-tetrazolium) or trypan blue staining assays), transfection efficiency (e.g., internalization of fluorescent nanoparticles/nucleic acids by confocal microscopy or/and flow cytometry), gene expression (e.g., RT-PCR, western blot, or ELISA assays), and sometimes functional assays (e.g., evaluation of cell proliferation, migration and invasion, colony formation, angiogenesis, and apoptosis), in vivo reports in animal models (mainly rodents) are mostly limited to measuring a therapeutic effect in terms of tumor growth, providing only a yes or no answer. Therefore, the causes behind the therapeutic failure are not well understood. In our opinion, it is necessary to learn more about the in vivo performance of genetic nanomedicines, and to incorporate functional assays in animal models, in order to speed up the translation of genetic nanomedicines to a clinical setting. Novel tools and models that would allow fast and low-cost comparative studies for the rational optimization of genetic nanomedicines are urgently needed.

## 3. Zebrafish as a Model Species

Zebrafish (*Danio rerio*) is a freshwater fish belonging to the *Cyprinidae* family, common in the river Ganga basin on the Indian sub-continent. Zebrafish has some well-known characteristics that makes it really attractive as a model for human diseases [62,63,64,65]. In fact, it has achieved the status of model species, and been presented as an extraordinary complement to murine models, and a promising alternative [64]. For one, zebrafish’s maintenance is affordable in terms of feasibility and costs. Moreover, adult individuals are small in size (2.5–4 cm), which makes the space requirements not very demanding. In addition, it has high fecundity and fertilization rates (up to 200 fertilized eggs per mating pair and week), and presents external fertilization, which allows for performing directed crosses, as well as in vitro fertilization. It also presents relatively short generation times—around three months. Finally, the genome of zebrafish, whose complete DNA sequence was published in 2013 [66], shows approximately 70% of homology with the human genome, and 82% of orthologous human disease-related genes.

Zebrafish embryos are particularly interesting for biomedical applications [67,68]. As early as 48 h post fertilization (hpf), embryos raised at 28.5 °C hatch from the chorion (external and acellular protective membrane), and become free-living animals with a complete body pattern, and almost completely functional organs [69]. At this time, the innate immune system is already active [70], but the adaptive immune system will not be fully operating until 4–6 weeks post fertilization (wpf) [71], although expression of some genes of the adaptive immune system starts as early as eight days post fertilization (dpf) [72]. Therefore, the results of analyses carried out during the embryo-larval phases can be traced back to the innate immune system.

Zebrafish embryos are robust and can survive different procedures right after fertilization, including genetic manipulation, morpholino [73,74,75] or ribonucleoprotein (CRISPR/Cas) [76,77,78,79] microinjection at single cell stage, as well as cancer cell xenotransplants [80,81,82,83,84,85]. In addition, they are transparent, which gives them a definite advantage in many fields of study, because it makes possible, for example, to examine the development of internal structures, and the tracking of the movements and biodistribution of labeled particles (microorganisms, cells, nanoparticles…) in real time [85,86,87,88]. Visualization can be hampered by the early production of melanin during their embryonic development, as early as 24 hpf (prim5 developmental stage). However, melanin production can be easily blocked by treating the embryos with 1-phenyl 2-thiourea (PTU) [69]. Additionally, the small size of zebrafish embryos (assays can be performed in 96 or, less suitably, in 384 multi-well plates), and the fact that they can live in small volumes (so that low quantities of the tested compounds are required) make this a suitable model for high-throughput analyses [89,90]. An adult also transparent line (*casper*) was developed [91], which allows for carrying out similar analyses in adults [92,93,94].

Finally, the European Food Safety Administration [95] has stated that fish in these early developmental stages, up to 5 dpf, are less likely to experience pain, suffering, distress, or suffer lasting harm, in accordance with the 3Rs Principles (replacement, reduction, and refinement) for humane animal research [96].

Therefore, taking all these facts into consideration, zebrafish has been accepted as a suitable model for biomedical purposes, for it could provide results faster than research on non-transparent, less prolific, more time-consuming, and expensive rodents, and improve the biological interpretation of the results compared to working on invertebrate models, which are phylogenetically further from human beings, and from in vitro analyses, which lack body interactions.

## 4. Zebrafish Is Currently Being Used for the Development of Anticancer Therapeutics

The pathological mechanisms underlying cancer are some of the most challenging processes to understand because of their variety and complexity. Zebrafish is considered a complementary model to murine and other previous models for the study of the genetic basis of cancer and for the evaluation of carcinogenic and novel antitumoral compounds in drug discovery [97,98,99,100,101,102,103,104,105,106,107,108].

Zebrafish has proven to be a good model to predict adverse drug effects during animal preclinical and human clinical data [109]. This is because many of the cellular and molecular mechanisms involved in zebrafish’s response to toxicity or stress are similar to those of mammals [110,111]. The publication of the DNA sequence of the zebrafish genome confirmed that relevant molecular pathways, including those implicated in cancer, are similar to those of mammals [66], which made zebrafish an attractive choice for cancer research [67,106,112,113]. A parallel approach for modeling cancer has been the (xeno) transplant of human cancer cells into zebrafish embryos, which led to the development of the so-called xenografted embryos. The proliferation, spreading and metastasizing of microinjected cancer cells is possible because the zebrafish embryos lack an adaptive immune system. Since the first successful model in 2005 and further improvements in 2006 [80,114], different xenograft zebrafish models have been reported bearing either commercial human cancer cell lines or primary tumor cells, including cancers from different origins (i.e., melanoma, breast carcinoma, colorectal, pancreatic, ovarian, kidney, lung, oral, prostate, leukemia, etc.) [80,82,85,115,116,117,118,119].

**Figure 1:**
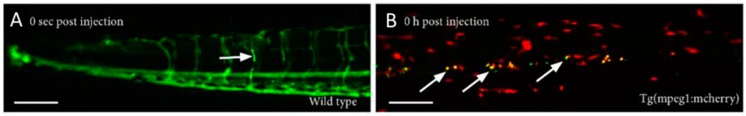
Green-labeled liposomes, injected into the circulatory system of wild type zebrafish embryos (**A**), allows the visualization of the fluorescent liposomes in the fish vasculature. On the right, the tg (mpeg1mecherry) model (**B**) shows the uptake of the fluorescent green liposomes by fluorescent red circulating macrophages (yellow dots). Imaging adapted from the work of Evensen et al. [127] with permission.

As indicated above, zebrafish cancer models have been used for novel drug screening, as well as for reanalysis of known drugs [97,100,105,106,107,108,113,120]. Nevertheless, due to the nanotechnology revolution on anticancer drug delivery, as stated in Section 1, recent studies also highlight the potential of zebrafish for the evaluation of novel anticancer nanomedicines. Most studies measured the toxicity and safety of blank nanoparticles (i.e., prior to drug incorporation) using different procedures, but also covered morphological descriptions of zebrafish after administration of sub lethal doses, and experiments of gene expression [68,121,122,123]. Taking advantage of the embryo transparency, biodistribution studies have also been performed to determine the ability of the nanocarriers to reach the target site, and even surpass complex biological barriers, such as the blood–brain barrier [124,125,126]. Apart from determining these critical parameters, the zebrafish xenograft model has also been proven useful in the study of the interaction between drug-loaded nanocarriers and xenografted cells, for example when studying a possible reduction in the population of cancer cells [68,127,128,129]. Among others, it is worth mentioning Yang and collaborators’ studies [129] that describe the interaction of targeted doxorubicin-loaded liposomes with HeLa cells, and the efficiency of this strategy in a xenograft model of zebrafish, and also the work of Evensen and collaborators [127] that describes the ability of PEGylated nanocarriers to avoid uptake by macrophages, a fact that translates in improved circulation times and increased accumulation into the tumors. Figure 1 depicts a visual example of liposomes labeled in green and distributed along the fish blood vessels upon injection into the circulation (A) and their subsequent uptake by macrophages labeled in red (yellow dots).

## 5. The Potential of Zebrafish for Increasing the Translation of Genetic Anticancer Nanomedicines: Barriers and Models

Apart from the use of zebrafish for the development of novel cancer therapeutics, including nanotherapeutics, only a few studies have been reported using this model to test preclinical genetic nanomedicines [130,131,132]. The first study found in the literature evaluates a synergistic therapy based on the co-encapsulation of a pigment-epithelium-derived factor (PEDF) plasmid with paclitaxel, a small molecular chemotherapeutic drug, into poly(lactic-co-glycolic acid) (PLGA) nanoparticles, in a transgenic zebrafish model Flk-1:eGFP. The results showed an active targeting that translates into an effective and safe antiangiogenic therapy [130]. The second example covers the development of a retro-inverse amphipathic RICK (retro-inverse form of the CADY-K peptide) peptide as novel non-covalent siRNA carrier. The designed nanoparticles show an effective siRNA protection, based on the specific protease resistant peptide sequence. The authors investigated the effect of a polyethylene glycol (PEG) grafting to RICK nanoparticles on their in vitro and in vivo capacity to deliver siRNA. In vivo assays performed in Casper zebrafish followed the biodistribution of fluorescent-labeled nanoparticles after injection at the one-cell stage in zebrafish embryos. The authors described a modular, easy-to-handle drug delivery system that could be adapted to other types of functional moieties in order to develop safe and biocompatible delivery systems for the clinical application of RNAi-based cancer therapeutics [131]. Finally, Cordeiro et al. [132] reported the design of a gold nanobeacon able to silence enhanced green fluorescence protein (EGFP) in embryos of a fli-EGFP transgenic zebrafish line. Results in this model allowed the authors to conclude that they have developed a biocompatible and efficient nanoplatform for gene silencing purposes.

**Figure 2:**
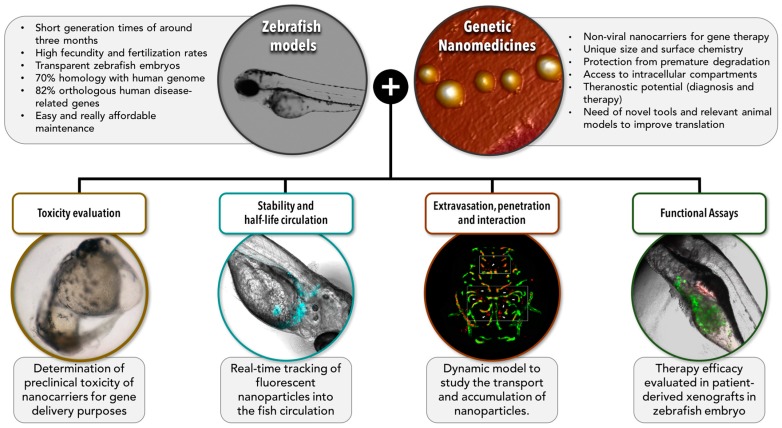
Zebrafish as a model organism for preclinical studies of genetic nanomedicines. This scheme highlights the main characteristics of zebrafish as model organisms and the main advantages of nanomedicines for gene delivery. The scope of this review is summarized in the lower section of the figure where we have illustrated different ways in which zebrafish models can be extremely useful to help us understand the biological behaviour of genetic nanomedicines, and define better prototypes with improved opportunities of translation to a clinical setting. Zebrafish models would allow performing several assays of interest such as (i) evaluation of the toxicological profile, (ii) determination of the stability and half-life circulation of nanomedicines inyected in the fish circulation system, (iii) study of the ability of nanomedicines to extravasate, difuse, penetrate into the tumor, and interact with the targeted cells, and (iv) functional assays to test the potential and the efficacy of the proposed nanomedicines. The two images on top correspond to a zebrafish embryo (left), and to nanometric (~100 nm) lipidic nanoemulsions observed by atomic force microscopy (AFM) (right). Images in the low part of the figure correspond, from left to right, to 48 hpf malformed zebrafish embryo due to toxic effects of nanocapsules (image reproduced with permission from Teijeiro-Valiño et al. [88], fluorescent DiD-labelled lipidic nanoemulsions (blue) injected into the fish circulation system and observed under a fluorescence microscope (images adquired at 48 h post-injection), fluorescent nanoparticles (red) able to extravasate blood vessels (green) in a zebrafish model (image obtained by confocal microscopy by Zou et al. [133], and reproduced with permission), and fluorescent DiD-labelled lipidic nanoemulsions (red) able to interact with cancer cells (green) in xenotransplanted zebrafish embryos (HCT116-GFP) after yolk microinjection.

As illustrated in Figure 2, a closer evaluation of the in vivo performance of genetic nanomedicines and a detailed study of their ability to overcome the critical barriers that might hamper a successful therapy are key factors in order to speed up their translation to clinic.

Next, we describe the most relevant barriers to gene delivery, and the zebrafish models that, in our understanding, can be useful for a rational design of successful anticancer genetic nanomedicines (compiled in Table 2).

### 5.1. Toxicity

Despite the ability of the nanoparticles to reduce the side effects of the associated drugs, adverse effects due to the nanoparticles themselves have been reported in some clinical studies, including immunotoxicity (allergy, hyper-sensitivity, and immunosuppression), acute toxicity (i.e., single-dose studies), subacute toxicity (i.e., repeated-dose studies or semi-chronic toxicity studies), carcinogenicity, reproductive toxicity, developmental toxicity, genotoxicity, hepatotoxicity or epigenotoxicity [134,135,136,137,138,139]. Nanoparticles may also activate innate immunity responses in the body and, as a consequence, they can mediate an uncontrolled delivery of pro-inflammatory mediators (anaphylatoxins) that could nullify the therapeutic effect of the nanocarrier and, even worse, promote tumor growth [140]. In the case of genetic nanomedicines, they typically contain cationic elements to improve their association with the anionic nucleic acids. These positively charged biomaterials have also been related to toxicity and off-target unspecific effects after transfection. Toxicity in preclinical studies relies mainly on simple and conventional tests (e.g., MTT assay), and, in some cases, systemic toxicity in vivo (e.g., serological and biochemical analysis of blood samples in mice). Therefore, it is clear that toxicity needs further attention before we can proceed to clinical studies.

As mentioned in Section 4, zebrafish is widely used for the evaluation of the adverse effects of drugs, and to determine the activity of antitumor compounds [97,98,99,100,101,102,103,104,105,106,107,108]. It could also be used to determine the preclinical toxicity of nanocarriers for gene delivery purposes. The most common and simple toxicity studies in wild type zebrafish relate to acute and chronic effects. Protocols for these studies have already been approved by the Organization for Economic Co-operation and Development (OECD). To determine zebrafish embryo toxicity, post fertilization embryos are placed in a static plate and exposed to the compound. The rate of morphological changes is one of the endpoints used to generate dose response curves [141,142]. The toxicity of several types of nanoparticles, mainly inorganic nanoparticles, has already been determined in zebrafish using this test [68,104,127,143,144]. One important parameter for toxicity evaluation is the hatching efficiency because nanoparticles can interact with hatching enzymes [145]. Zebrafish is also a versatile organism for genotoxicity studies [146,147,148,149], developmental and behavioral analysis [150,151,152,153], immunotoxicity [154,155], neurotoxicity [156,157], and reproductive toxicity studies [158]. For example, in experiments with transgenic lines, such as Tg(flk1:eGFP), Tg(cmlc2:eGFP), Hsp70:eGFP, ARE:eGFP, FLI-1, and Nacre/fli1:EGFP, it was possible to observe the chemical-induced toxicity of nanocomposites and metal oxide nanoparticles in real time [159,160,161,162,163].

Zebrafish is also an excellent model to provide novel insights on the interaction between the immune system and tumor cells [164,165]. Because in zebrafish, macrophages play an important role in angiogenesis, this model could also be used to develop functional assays related to the angiogenic process (Section 5.4). A transgenic zebrafish line, mpo:GFP, which expresses GFP under the neutrophil-specific myeloperoxidase promoter, has also been described and used to study neutrophil response [166], including the evaluation of oxidative stress and inflammatory responses in neutrophils following the administration of silica nanoparticles [167]. In addition, studies regarding cardiotoxicity are also of great importance, among them is worth mentioning the evaluation of effects occurring immediately after administration and their consequences [153].

### 5.2. Stability and Half-Life While in Circulation

Preclinical studies sometimes ignore the fact that the electrostatic stability of nanocarriers in vitro does not guarantee their stability in vivo. Moreover, in many cases, the nanocarrier and the gene vector are associated by electrostatic interactions. Upon contact with a biological media of high ionic strength, this system may aggregate, resulting in the displacement of the nucleic acids that could be prematurely released into the circulation before reaching the target cells. The presence of serum proteins (e.g., glycosaminoglycans) could have the same effect. Therefore, a thorough study, relevant in vivo models, of the stability and interactions of the nanocarrier under study could be necessary to ensure that the associated nucleic acids are not prematurely released into the circulation [168,169,170]. On the other hand, nanosystems should also be able to avoid recognition by macrophages, and a rapid clearance by the mononuclear phagocyte system (MPS), which would lead to their fast removal from circulation [136].

As stated in Section 2, one of the main advantages of zebrafish embryos and adults from the Casper line is that they are transparent, and therefore suitable for direct and real-time tracking of fluorescent nanoparticles into the fish circulation, using high-resolution confocal microscopy [91]. Importantly, a recent study shows a good correlation among pharmacokinetic data obtained in zebrafish, rat, and mice, and highlights the potential of zebrafish for this purpose [126]. Different studies carried out with model nanoparticles, FluoSpheres^®^ and Quantum Dots^®^, highlight the influence of the exposure route (waterborne, injection and oral), and surface properties of the nanoparticles on their biodistribution and tumor uptake [35,171].

One model useful for tracking the circulation of nanoparticles is the transgenic line Fli1:eGFP [127]. This line has allowed for following the distribution and tumor accumulation of PEGylated nanoparticles. In the same study, the Tg(mpeg1:mCherry) line was selected to evaluate the interaction of these nanoparticles with macrophages, which led to the conclusion that PEG coating actually decreased the interaction of the nanoparticles with macrophages. Transgenic lines of macrophages, neutrophils, and endothelial cells expressing fluorescent markers (see Table 2) have also been used to watch the interaction between lipid nanoparticles and immune cells [87].

### 5.3. Extravasation, Penetration into the Tumor, and Interaction with the Target Cells

Nanocarriers should be able to exit the systemic circulation at the action site. Recently, it has been reported that current animal models fail to predict the accumulation of nanocarriers inside the tumor, which is actually about 0.7% of the injected dose [172,173]. Thus, animal models that would allow us to better study the ability of nanocarriers in this step are crucial to ensuring an effective therapeutic effect [174]. The complexity of the tumor extracellular matrix (ECM) may also restrict the extravasation of the nanocarriers. Additionally, even if the nanocarriers could cross the tumor vasculature, they might not be able to penetrate deep enough inside the tumor mass due to the high interstitial fluid pressure, and might accumulate instead in the peripheral areas, or in the surrounding healthy tissue [15,175]. Finally, the nanoparticles need to interact with the target cells. Typically, therapies are directed at tumor cells, but they can also be designed to target cells of the stroma or to infiltrate immune cells, cancer stem cells (CSCs), cancer-associated fibroblasts (CAFs), tumor-associated macrophages (TAMs), pericytes, endothelial cells, etc. [15,176].

To date, an extensive list of improved zebrafish cancer models has been reported, including models to study neuroblastoma, brain cancer, eye cancer, leukemia, melanoma, uveal melanoma, and liver cancer, among others [177]. More complex models to study the mechanisms of tumor cell dissemination and metastases formation have also been reported [178,179]. For example, the model Flk1:EGFP has been used to study the metastatic spread after injection of red fluorescent protein (RFP)-labeled Hela cells in the caudal artery [180]. Other results show how metastatic cell lines have improved abilities to migrate and proliferate compared to cells isolated from primary tumors [181]. The study of CSC has also been considered in zebrafish models [182,183]. Regarding the study of the tumor microenvironment (TME), Zhao et al. [184] showed that transforming growth factor beta (TGF-β) induced a pro-tumor neutrophil cytokine expression pattern in zebrafish, and concluded that essential mechanisms in the constitution of the TME are conserved in this model.

Regarding the particular evaluation of nanomedicines, several works cover the evaluation of their ability to accumulate in tumor cells after injection in zebrafish xenografts [125,127,185,186,187]. Zebrafish can therefore be considered as a dynamic model to study the transport and accumulation of nanoparticles.

### 5.4. Functional Assays

We believe that, when performing functional assays, zebrafish models are very useful for determining the efficacy of the therapy. Importantly, it is feasible to use xenografts of patient-derived tumor cells in zebrafish embryos, to perform patient-specific drug screens, and analyze critical aspects of the tumor, such as growth and proliferation [80], invasion and intravasation [180,188,189], formation of metastasis [82,190], angiogenesis [190,191], and immune cell response [107]. Hundreds of embryos can be injected in a single day, and it is possible to exploit the imaging capabilities of the zebrafish. Cell injections in fish can be performed in the duct of Cuvier, vein, and yolk sac, as well as pericardially, intracardiaally, and in the brain parenchyma, in order to obtain different read-outs. For example, since the yolk sac does not communicate with the vasculature directly, it would be a good model to study metastasis by either invasion or blood borne spreading [118]. Additionally, to study specific phenomena such as angiogenesis, there are useful transgenic lines, such as Tg(Flk1:EGFP) and Tg(Fli1:EGFP) with green vasculature, and Tg(Gata1:DsRed) with red fluorescent blood cells [107]. These models allow the study of the distribution and functionalities of nanoscale drug delivery systems [180]. As an example, one study used curcumine-loaded micelles to test the potential of zebrafish for developing novel anti-angiogenic and antitumoral therapies [187]. In a different work using silica nanoparticles, it was possible to observe inhibition of angiogenesis via vascular endothelial growth factor receptor 2 (VEGFR2)-mediated mitogen-activated protein kinase (MAPK) signaling pathway [192]. Other authors have claimed a reduction in the number of tumor cells transplanted into fish, upon delivery of anti-tumor nanomedicines [127,185,186]. Additionally, it would be possible to determine whether nanoparticles carrying the proposed therapy induce apoptosis: a fluorescent probe designed to characterize patterns of apoptosis in living zebrafish larvae has recently been described [193].

## 6. Conclusions

We have summarized here the main advances in the field of cancer nanomedicine, and the increasing interest in the nanotechnology field for the development of safe and efficient anticancer gene therapies, and highlighted the main limitations of this approach. We have described several models of zebrafish and discussed assays that, to date, have been applied mainly for other purposes, such as in cancer biology, toxicology, and drug screening studies, but that, in our opinion, hold an enormous potential for speeding up the translation of genetic nanomedicines for cancer treatment. We are confident that, in the next few years, great advances in the fight against cancer will be made thanks to this versatile animal model.

## Figures and Tables

**Table 1:** Main studies to date of genetic nanomedicines that have had relevant therapeutic effects on different types of cancer in mice models.

Nanocarrier	Gene Vector	Target	Indication	Administration Route	Ref
Liposomes	miRNA	Restoration of oncossuppressor	Breast cancer	Tail vein	[40]
	siRNA	EpCAM silencing	Breast cancer	Tumor adjacent	[41]
	siRNA	Anti-angiogenesis	Breast cancer	Intratumoral	[42]
	miRNA	Restoration of oncosuppressor	Hepatocellular carcinoma	Intratumoral	[43]
	shRNA	WT1 silencing	Melanoma	Tail vein	[44]
Polymeric nanoparticles	pDNA	Anti-angiogenesis	Colon cancer	Tail vein	[45]
	pDNA	Induce apoptosis	Ovarian cancer	Intraperitoneal	[46]
	pDNA	Suicide gene therapy	Ovarian cancer	Intraperitoneal	[47]
	pDNA	Immunotherapy	Colorectal cancer	Intratumoral	[48]
	pDNA	Suicide gene therapy	Colon cancer	Intratumoral	[49]
Lipid nanoparticles	siRNA	Androgen receptor silencing	Prostate cancer	Tail vein	[50]
	miRNA	Restoration of microRNA-26a	Lymphocytic leukemia	Intraperitoneal	[51]
Dendrimers	si/shRNA	ITCH silencing	Pancreatic cancer	Tail vein	[52]

siRNA, small interference RNA; shRNA, short-hairpin RNA; pDNA, plasmid DNA; miRNA, microRNA; EpCAM, epithelial cell adhesion molecule; WT1, Wilms Tumor 1.

**Table 2:** Selected zebrafish models of potential interest for the biological evaluation of genetic nanomedicines.

Model	Features	Application	Ref
Wild type	From nature, with pigmentation according to sex, without fluorescence	Toxicity, biodistribution, xenograft	[194]
Flk-1:eGFP	Fluorescent vascular system	Toxicity, biodistribution, xenograft, angiogenesis, extravasation, half-life circulation, metastasis	[107,130]
Fli-1:eGFP			[107,127,162]
Gata1:DsRed			[107]
Nacre/fli1:eGFP			[163]
Casper fli	Without pigmentation (transparent) and fluorescent vascular system		[91]
Casper	Without pigmentation (transparent)	Toxicity, biodistribution, xenograft, metastasis	[91]
ARE:eGFP	Fluorescence of reactive oxygen species (ROS)	Toxicity	[162]
Cmlc2:eGFP	Fluorescence in the heart	Cardiotoxicity	[167]
Mpo:GFP	Fluorescent neutrophils	Interaction, half-life circulation, immuno response	[167]
Mpeg1:mcherry	Fluorescent macrophages		[127]
Hsp70:eGFP	Fluorescence of the protein HSP70 stress product	Toxicity	[195]
